# Efficacy of Acupuncture Treatment of Migraine Delivered by Senior or Junior Acupuncturists: Study Protocol for a Randomized Controlled Trial

**DOI:** 10.3389/fneur.2021.812504

**Published:** 2022-02-07

**Authors:** Jun Zhou, Nan-nan Jiang, Yu Fang, Xin-yue Zhang, Shi-rui Cheng, Xin-ling Li, Sheng-jie Hu, Rui-rui Sun, Hua-bin Zheng, Xiao-peng Huang, Fang Zeng, Fan-rong Liang, Zheng-jie Li

**Affiliations:** ^1^Acupuncture and Tuina School/The 3rd Teaching Hospital, Chengdu University of Traditional Chinese Medicine, Chengdu, China; ^2^Neurology Department, Hospital of Chengdu University of Traditional Chinese Medicine, Chengdu, China; ^3^Acupuncture and Brain Research Center, Chengdu University of Traditional Chinese Medicine, Chengdu, China

**Keywords:** acupuncturist, clinical effect, expectation, migraine, RCT

## Abstract

**Introduction:**

Acupuncture is an efficacious and safe treatment choice for migraine prevention. Results from clinical trials have shown that non-specific effects play an important role in acupuncture's efficacy. To date, however, there is no evidence available quantitatively evaluating the effect of non-specific effects, such as patients' expectations and beliefs for acupuncturists, on acupuncture efficacy, necessitating further exploration.

**Methods:**

A total of 156 patients with migraine without aura (MwoA) will be randomized to either junior or senior acupuncturist group, at a ratio of 1:1. The study will last 24 weeks, for each patient, comprising baseline, treatment, and follow-up phases lasting 4, 8, and 12 weeks, respectively. All patients will undergo 12 sections of acupuncture treatment delivered by either a junior or senior acupuncturist following the same acupuncture prescription and manipulation. The primary outcomes will be changes in the number of migraine days and frequency of attacks per 4 weeks cycle, relative to the baseline. Secondary outcomes will include severity of headache pain, quality of life, anxiety/depression levels, and use of non-steroidal anti-inflammatory drugs (NSAIDs) per 4 weeks cycle, compared to the baseline, as well as adverse events and rate of positive response to treatment. Prior to randomization of patients into junior or senior acupuncturist groups, the Acupuncture Expectations Evaluation Scale (AES) will be used to evaluate their expectations and belief with regards to acupuncture efficacy delivered by senior or junior acupuncturists.

**Discussion:**

Results from this clinical randomized controlled trial will help to quantitatively evaluate the extent of the effect of acupuncture treatment delivered by a senior or junior acupuncturist (high relative to low expectations) in migraine patients.

**Ethics and Dissemination:**

This trial has been approved by the Institutional Review Boards and Ethics Committees of Hospital of Chengdu University of Traditional Chinese Medicine (Approval No. 2020KL-058).

## Introduction

Migraine is a widespread neurological disorder that affects an estimated 1 billion people worldwide (1). Migraine has different subtypes, about 64% of which is migraine without aura (MwoA) subtype (2). According to the 2016 Global Burden of Disease Study, migraine is the second leading cause of disabilities, more than all other neurological disorders combined (3). Studies have estimated that in the UK alone, the direct and indirect costs associated with migraine are about £3.42 billion per year, taking into consideration disability, productivity loss, and healthcare costs (4). The current treatment therapies have not effectively managed migraine (3), necessitating exploration of additional effective, low-risk, and low-cost strategies.

Acupuncture, which is an important component of traditional Chinese medicine (TCM), has been used to treat headaches in East Asian countries for thousands of years. At present, it is also used for migraine treatment across many other regions worldwide, owing to its significant efficacy, few side effects, and cost-effectiveness (5–7). Results from numerous clinical trials have confirmed the immediate and long-term effects of acupuncture on migraine headaches and their corresponding symptoms, such as number of migraine days, headache frequency, and headache intensity (8–12). Although acupuncture is an effective and safe therapy for treatment of migraine, a recent report from Cochrane Database of Systematic Reviews revealed that non-specific effects play an important role in its efficacy (13). To date, however, little is known regarding to what extent the effects of acupuncture for migraine are mediated by context variables, such as patients' expectations of the acupuncturist, their preference for treatment choices, and culture background, among others. Therefore, further explorations are needed to elucidate the extent to which these factors affect acupuncture-based treatment of migraine.

In China, there is a popular proverb that says, “old TCM physician,” meaning that old- and high-level TCM physicians are more experienced. Therefore, patients prefer to consult senior and high-level TCM physicians for acupuncture treatment, because they believe that more experienced and high-level acupuncturists will bring them better therapeutic efficacy. However, in some cases, both senior and junior acupuncturists follow similar and standard acupuncture prescription and manipulation. Patients' expectation for the acupuncturist might influence the clinical efficacy. However, up to now, there is no evidence available quantitatively evaluating the extent of the effect of patients' expectation and confidence of an acupuncturist in acupuncture treatment for migraine.

Based on the above, in this study, we hypothesize that patients' expectation and confidence of an acupuncturist might have a potential impact on acupuncture efficacy. Therefore, this randomized controlled trial (RCT) aims to quantitatively evaluate the extent of the effect of acupuncture treatment delivered by either a senior (high expectation) or junior (low expectation) acupuncturist during treatment of migraine patients.

## Methods and Analysis

### Objective

To quantitatively evaluate the extent of the effect of acupuncture treatment delivered by either a senior (high expectation) or junior (low expectation) acupuncturist in migraine patients.

### Hypotheses

(1). Patients have higher expectations of a senior than a junior acupuncturist.
(2). Patients treated by a senior acupuncturist group exhibit moderately better therapeutic effect after acupuncture treatment than those in the junior acupuncturist group.

### Study Design

**Figure 1:**
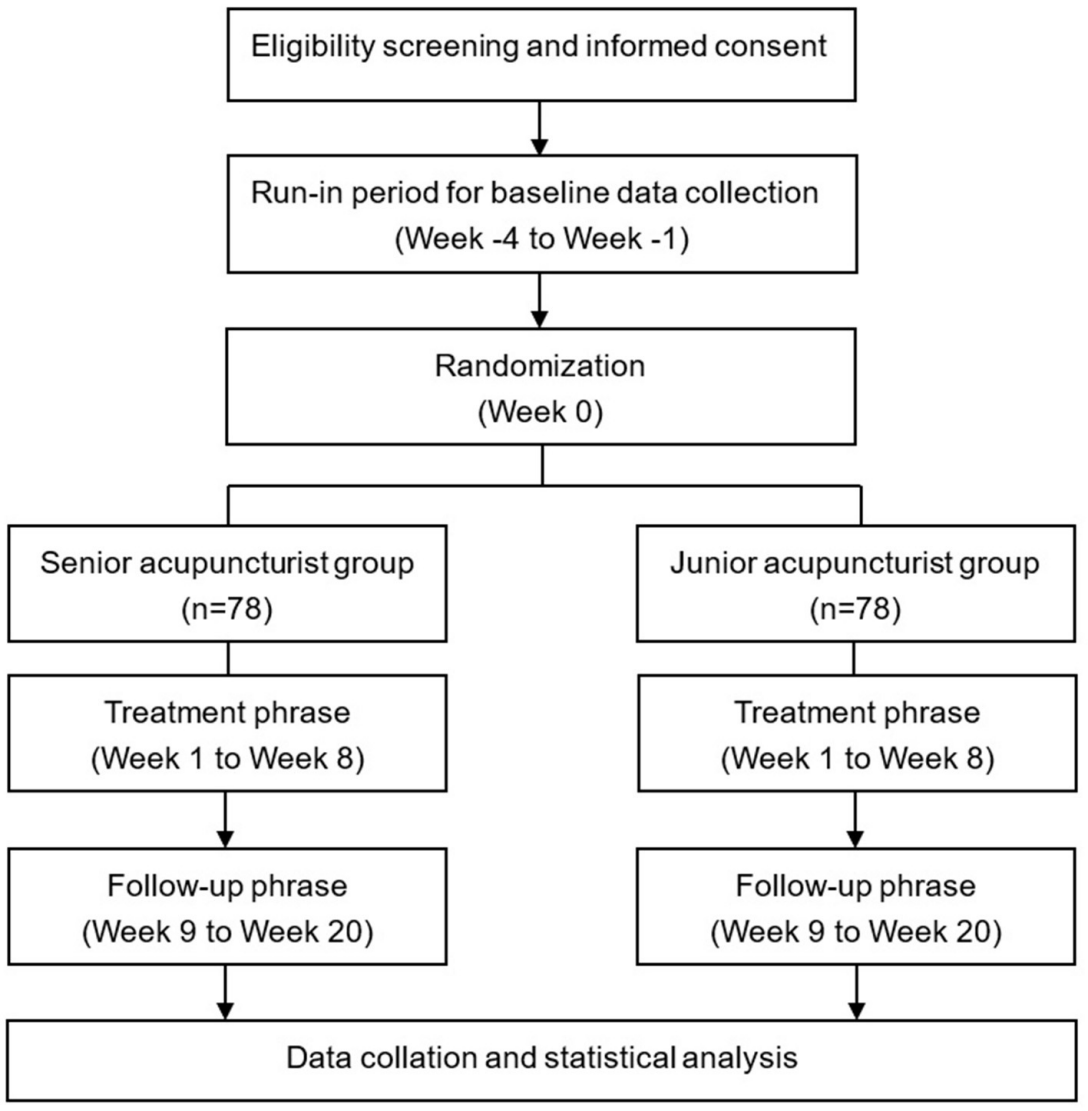
Study flowchart.

This will be a single-center, two parallel-group, randomized clinical trial. A total of 156 MwoA patients will be recruited, then randomly allocated to either senior or junior acupuncturist groups, at a ratio of 1:1. The study will be conducted for a period of 24 weeks for each patient, comprising the following phases: baseline (4 weeks), treatment (8 weeks), and follow-up (12 weeks). AES will be used to evaluate patients' expectations for acupuncture efficacy delivered by either a junior or senior acupuncturist before randomization. A summary of the study protocol is presented using a flowchart (Figure 1) while the schedule is outlined in Table 1. This protocol is reported in accordance with the guidelines of the Consolidated Standards of Reporting Trials (CONSORT) (14) and Standards for Reporting Interventions in Clinical Trials of Acupuncture (STRICTA) (15).

**Table 1:** Standard Protocol Items: Recommendations for Interventional Trials (SPIRIT) schedule of the trial.

	**STUDY PERIOD**								
	**Baseline**			**Treatment**			**Follow-up**		
**TIMEPOINT**	**-4-week**	**0-week**	**2-week**	**4-week**	**6-week**	**8-week**	**12-week**	**16-week**	**20-week**
**ENROLLMENT:**									
Eligibility screen	**×**								
Informed consent		**×**							
**INTERVENTION:**									
Senior acupuncturist acupuncture treatment			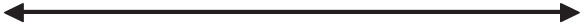						
Junior acupuncturist acupuncture treatment			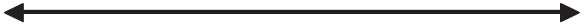						
**ASSESSMENTS:**									
The headache diary	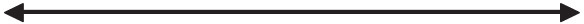								
Number of days with migraine	**×**	**×**		**×**		**×**	**×**	**×**	**×**
Frequency of migraine attacks	**×**	**×**		**×**		**×**	**×**	**×**	**×**
Positive response rate				**×**		**×**	**×**	**×**	**×**
Mean VAS score		**×**		**×**		**×**	**×**	**×**	**×**
SAS score		**×**		**×**		**×**	**×**	**×**	**×**
SDS score		**×**		**×**		**×**	**×**	**×**	**×**
The use of NSAIDs	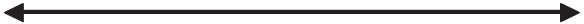								
MIDAS		**×**		**×**		**×**	**×**	**×**	**×**
MSQ		**×**		**×**		**×**	**×**	**×**	**×**
AES		**×**							
MASS			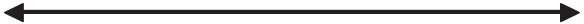						
**PARTICIPANTS SAFETY:**									
Laboratory test		**×**				**×**			
Adverse events	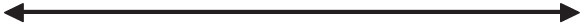								

*This study includes 4-week baseline period, 8-week treatment period and 12-week follow-up period for each migraine patient*.

*AES, acupuncture expectations evaluation scale; MASS, Massachusetts General Hospital acupuncture sensation scale; MIDAS, Migraine Disability Assessment Scale; MSQ, Migraine-Specific Quality-of-Life Questionnaire; NSAIDs, nonsteroidal anti-inflammatory drugs; SAS, self-rating anxiety scale; SDS, self-rating depression scale; VAS, visual analog scale; Laboratory test: including routine examination of blood, urine and stool, blood biochemical test (including liver and kidney function), and electrocardiogram examination*.

### Patient Recruitment

Potential MwoA patients are currently being recruited from outpatient clinics at the Hospital of Chengdu University of Traditional Chinese Medicine, local communities, and WeChat advertisements. The recruitment time started in March 2021 and will end in December 2023.

### Eligibility Criteria

All potential MwoA patients will be assessed face-to-face for eligibility by neurologists of Hospital of Chengdu University of Traditional Chinese Medicine according to the following inclusion and exclusion criteria.

#### Inclusion Criteria

Participants will be recruited if they:

(1) are aged between 18 to 65 years;
(2) are diagnosed with MwoA according to the international classification of headache disorders (ICHD-3) 2018 (16);
(3) have a history of MwoA for over 6 months;
(4) have had MwoA attacks, 2–8 times per month in the past 3 months. These attacks should have lasted 4–72 h without or at least 2 h with medication;
(5) voluntarily sign an informed consent.

#### Exclusion Criteria

Participants will be excluded if they:

(1) are accompanied with any other chronic pain conditions or have a history of head trauma (with loss of consciousness) or mental retardation;
(2) are suffering from psychiatric, respiratory, cardiovascular, or renal illnesses;
(3) have previously received acupuncture treatment, or taken any prophylactic headache medicine in the last 1 month;
(4) have a history of alcohol and/or other substance abuse or dependence;
(5) are carrying a pregnancy, ready to be pregnant, or lactating mothers;
(6) have acupuncture contraindications, such as bleeding tendency;
(7) are unable to cooperate with acupuncture treatments;
(8) are participating in other trials within 3 months before recruitment.

### Randomization and Allocation Concealment

Patients will be randomly assigned to either group A or B, using a computerized randomization sequence form according to the random number table method. Odd and even numbers will be allocated to the senior (group A) and junior (group B) acupuncturist groups, respectively. Sealed opaque envelopes, with patients screening sequence numbers and group names printed on the outside and inside, respectively, will be used for the allocation. All the recruited patients will be numbered consecutively and connected correspondingly to the envelopes. After the baseline observation phase, the envelopes will be opened, and patients will be randomly assigned to either of the two groups.

### Blinding

Patients and acupuncturists will not be blinded due to the design and purpose of this study. Data collectors, outcome assessors, and data analysts will be blinded to the allocation.

### Sample Size Estimation

There is no available reference of clinical trials regarding the effect of patients' expectations of different acupuncturists on acupuncture-based migraine treatment so far. Our clinical experience and results from preliminary experiments indicate that the reduction number of migraine days was 3.9 ± 2.49 in the senior acupuncturist group, compared to 2.7 ± 3.45 days in the junior acupuncturist group at weeks 4–8 of treatment. PASS software version 15 was used to calculate the sample size, based on 80% power to detect a significant difference (α = 0.05, two sided), and with an excepted dropout rate of ~20%, target enrollment was set at 156 patients (78 patients in each group).

### Intervention

All MwoA patients will undergo acupuncture treatment, delivered by either a senior or junior acupuncturist, following the same protocol of acupuncture prescription and manipulation. The acupuncture prescription will follow the guidelines of whole head headache treatment, described in the national textbook of Chinese Acupuncture and Moxibustion for TCM students in mainland China. This includes Baihui (GV20), bilateral Touwei (ST8), bilateral Shuaigu (GB8), bilateral Taiyang (EX-HN5), bilateral Fengchi (GB20), bilateral Hegu (LI4), and bilateral Taichong (LR3).

**Figure 2:**
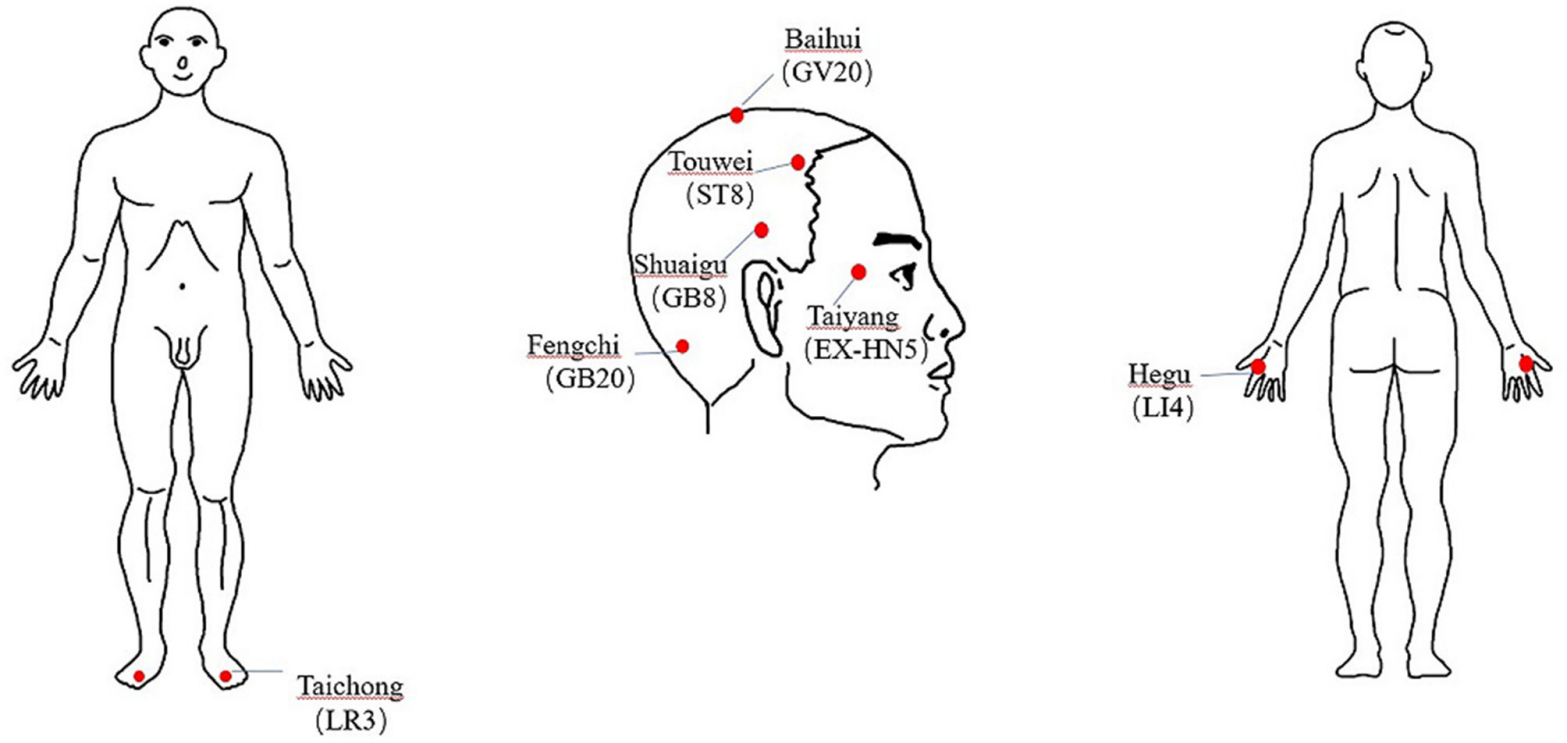
Indication of acupoints location of the acupuncture prescription in this study. The name, English code and location of each acupoint is in accordance with WHO Standard Acupuncture Point Locations in the Western Pacific Region. Retaining needles for 60 mins. Lifting, thrusting, twisting and/or rotating the needles once every 20 mins with intermittent stimulation for maintaining the deqi sensation for twice. EX-HN, Extrachannel points; GB, Gallbladder Meridian; GV, Governor Vessel; LI, Large Intestine Meridian; LR, Liver Meridian; ST, Stomach Meridian.

The acupuncture procedure will be as follows: local skin disinfection will first be performed, then acupuncture needles, with a diameter of 0.25 mm, and a length of 40 or 15 mm (Hwatuo, Suzhou, China), inserted perpendicularly, subcutaneously, or obliquely into the acupoints. After provocation of deqi (needle sensation) (17), the needles will be maintained therein for 60 min, with manipulation once every 20 min during this period, to maintain deqi sensation. Each MwoA patient will undergo 12 sessions of acupuncture treatment, lasting a period of 8 weeks. The treatment will be performed twice a week, for the first 4 weeks, and once a week for the next 4 weeks. All the acupuncture treatments in senior acupuncturist group will be delivered by a licensed senior acupuncturist with an associate professor title. On the other hand, procedures in the junior acupuncturist group will be delivered by a licensed junior acupuncturist with an intern title. Prior to commencement of the study, both acupuncturists were trained to follow the same acupuncture procedure to minimize differences in acupuncture manipulation parameters. See Table 2 and Figure 2 for details of the location and manipulation of the acupoints.

**Table 2:** Details of the acupoints location and insertion.

**Acupoints**	**Location**	**Depth of insertion**
*Baihui* (GV20)	On the head, 5 cun superior to the anterior hairline, on the anterior median line	0.5–0.8 cun (12.5–20 mm) subcutaneous insertion.
*Shuaigu* (GB8)	On the bilateral head, directly superior to the auricular apex, 1.5 cun superior to the temporal hairline.	0.5–0.8 cun (12.5–20 mm) subcutaneous insertion.
*Fengchi* (GB20)	In the anterior region of the neck, inferior to the occipital bone, in the depression between the origins of sternocleidomastoid and the trapezius muscles.	0.5–0.8 cun (12.5–20 mm) oblique insertion toward the tip of the nose.
*Touwei* (ST8)	On the bilateral head, 0.5 cun directly superior to the anterior hairline at the corner of the forehead, 4.5 cun lateral to the anterior median line.	0.5–0.8 cun (12.5–20 mm) subcutaneous insertion.
*Taiyang* (EX-HN5)	At the bilateral temporal part of the head, in the depression 1 cun posterior of the midpoint of the ligature between lateral end of the eyebrow and the outer canthus.	0.3–0.5 cun (7.5–12.5 mm) perpendicular insertion.
*Hegu* (LI4)	On the dorsum of the hand, radial to the midpoint of the second metacarpal bone.	0.5–0.8 cun (12.5–20 mm) perpendicular insertion.
*Taichong* (LR3)	On the dorsum of the foot, between the first and second metatarsal bones, in the depression distal to the junction of the bases of the two bones, over the dorsalis pedis artery.	0.5–0.8 cun (12.5–20 mm) perpendicular insertion.

*The name, English code and location of each acupoint is in accordance with WHO Standard Acupuncture Point Locations in the Western Pacific Region. Retaining needles for 60 mins. Lifting, thrusting, twisting and/or rotating the needles once every 20 mins with intermittent stimulation for maintaining the deqi sensation for twice. Cun belongs to proportional measurement unit for acupoints locating. EX-HN, Extrachannel points; GB, Gallbladder Meridian; GV, Governor Vessel; LI, Large Intestine Meridian; LR, Liver Meridian; ST, Stomach Meridian*.

### Cointerventions

Patients will be advised not to undergo any additional treatments for their migraine, and ibuprofen (300 mg each capsule with sustained release) will be recommended for management of severe pain, according to the evidence-based clinical practice guidelines (Scottish Intercollegiate Guidelines Network, 2018) (18). In intolerance or unresponsive to ibuprofen cases, other tolerable and responsive non-steroidal anti-inflammatory drugs (NSAIDs) could be used. The patients will be asked to record the name and dosage of any co-interventions they use during this clinical trial in a headache diary.

### Patient Safety

Routine examination of basic parameters, such as liver and renal function, as well as blood, urine, and feces will be performed in all patients prior to and after acupuncture treatment. Moreover, electrocardiography tests will be conducted prior to treatment, while any side effects following acupuncture will be descriptively recorded in the Case Report Form (CRF). Severe adverse events will also be monitored, managed, followed up, and recorded as previously described. These will be reported to the Ethics Committee within 24 h of occurrence.

### Outcome Measures

All MwoA patients will be asked to keep a headache diary during the whole 24-week observation period. The following endpoints will be assessed: (1) primary endpoint, namely number of migraine days and attacks; (2) secondary endpoints, namely response rates, severity of headache pain, quality of life, anxiety/depression of the patients, NSAIDs use, and adverse events; (3) other endpoints will include needle sensation evaluations and the patient's expectations for the acupuncturist. To ensure reliability of the results, all outcome data will be independently collected by trained assessors, and the acupuncturist will not be present during evaluation. Details on measurement of outcomes and the time of data collection are described below and in Table 1.

#### Primary Outcome Measurement

The primary outcomes will comprise changes in the mean number of migraine days and the frequency of migraine attacks per 4 weeks cycle, compared to those at baseline.

### Secondary Outcome Measurement

#### Positive Response Rate

A positive responder is a patient that exhibits at least 50% reduction in migraine days or frequency (19). The proportion of positive responders will be counted every 4 weeks, from baseline to endpoints.

#### Headache Severity

The intensity of headache will be assessed using the mean visual analog scale (VAS), which comprises a range of 0–10 points (20). This will be done from baseline to endpoints.

#### Emotional Status

Anxiety and depression symptoms will be assessed using the *Zung* Self-rating Anxiety Scale (*Zung* SAS) (21) and *Zung* Self-rating Depression Scale (*Zung* SDS) (22), respectively. Both scales have a 20-item self-reporting questionnaire, which are scored on a 4-point Likert scale (range 0–4 points) according to the frequency of symptoms in the past 4 weeks. A high SAS or SDS score (≥50) indicates severe anxiety or depression, respectively.

#### Quality of Life

We will employ the Migraine-Specific Quality-of-life Questionnaire (MSQ) (23), a validated and widely accepted tool that measures health-related quality of life (24). The MSQ measures a person's functional level, with higher scores implying better function. The Migraine Disability Assessment Scale (MIDAS) (25), a five-item questionnaire developed to measure headache-related disability that reveals the impact of migraine on daily functioning over the past three months, will also be used. MIDAS total scores range from 0–27 points, while MIDAS grades are defined as follows: I (0–5), II (6–10), III (11–20), and IV (>20), of which grade IV indicates the highest disability. Changes in MSQ and MIDAS scores will be measured every 4 weeks, from baseline to endpoints.

#### Use of NSAIDs

Ibuprofen (300 mg each capsule with sustained release) will be recommended as rescue medication, in cases who experience intolerable pain. In cases of intolerance or unresponsive to ibuprofen, other tolerable and unresponsive non-steroidal anti-inflammatory drugs (NSAIDs) could be used. The patients will be asked to record the name and dosage of NSAIDs they use during the course of the clinical trial in a headache diary.

#### Adverse Events

Acupuncture-related side effects, including serious pain, bleeding, subcutaneous hemorrhage, fainting, palpitation, and local infections, among others, will be recorded after each treatment. Additionally, adverse reactions to cointerventions will also be recorded in detail in the CRF during the whole study.

### Measurement of Other Outcomes

#### Needle Sensation Evaluations

Needle sensation (“deqi”) will be evaluated using the Massachusetts General Hospital (MGH) Acupuncture Sensation Scale (MASS) (26), a rubric designed for measurement of sensations evoked by acupuncture stimulation as perceived by the patient alone. Notably, patients will be required to fill in the MASS after each session of acupuncture treatment. This MASS will then be used, as a covariate, to exclude the potential influence of different needle sensation caused by different acupuncturists on the efficacy of acupuncture.

#### Evaluation of Patient's Expectations

The patient's expectation will be measured by AES (Appendix 1), including the expectation of the acupuncturist and the expectation of acupuncture therapy. Each patient will be asked to rate their expectation and confidence level for a junior and senior acupuncturist, respectively, using two opposing questions before randomization. The questions are as follows: “How much confidence do you have in senior acupuncturists to relieve your migraine symptoms?” and “How much confidence do you have in junior acupuncturists to relieve your migraine symptoms?” Each question adopts the percentile system, which is scored as summary percentile scales ranging from 0 to 100. Higher scores imply greater expectation and confidence. Besides, the other item of AES will be used to evaluate the patient's expectation of acupuncture therapy as a covariate.

### Statistical Analysis

Intention-to-treat analyses will be performed where possible, with subgroup and sensitivity analyses performed, where necessary, to detect possible heterogeneity of the results. All data will be statistically analyzed using SPSS version 26.0 (SPSS Inc., Chicago, IL, USA). Two-tailed analyses will be conducted, with the level of statistical significance defined set at *P* < 0.05. Data from normally distributed continuous variables will be expressed as means with their respective standard deviations (SD), unless otherwise specified, whereas categorical variables will be presented as numbers and percentages. Distribution of continuous variables will first be analyzed using the Shapiro-Wilk test and normal probability plot. Next, we will perform a Student's *t*-test to determine differences between groups for normally distributed data. The nonparametric test will be used for skewed data, or on data with heterogeneity of variance or unknown distribution. Comparisons in response rates and incidence rate of adverse events between groups will be performed by Chi-square or Fisher's exact tests.

## Discussion

This trial will adopt a two parallel-group randomized controlled trial design. To our knowledge, this is the first RCT seeking to quantitatively evaluate the effect of patients' expectations of an acupuncturist on the efficacy of acupuncture treatment for migraine. The findings of this trial are expected to help preliminarily evaluate the extent of patients' confidence and trust in efficacy of acupuncture treatment delivered by a senior or junior acupuncturist.

### Non-specific Effect Plays an Important Role in Acupuncture Analgesia

Acupuncture has long been considered a magic placebo trick for the management of chronic pain conditions. Nowadays, results from accumulating high quality clinical evidences have supported the use of acupuncture for pain relief (27–30). In fact, this therapy is now widely accepted as a safe and efficacious strategy for migraine prevention, but the effect of verum over sham acupuncture is only moderate (13, 31). This finding has been corroborated by results from several large-scale meta-analyses targeting acupuncture treatment for chronic pain conditions (32–34). It is undeniable that placebo effect (psychological effect) is an important part of acupuncture analgesia (28). Results from a psychological study showed that both the relationship between intervention measures and patients as well as that between therapists and patients significantly influences clinical efficacy (35). Moreover, results from several randomized controlled trials (RCTs), analyzing the use of acupuncture for treatment of chronic pain, found that the expectation of relief by acupuncture treatment could correctly predict outcomes (36). To date, however, little is known regarding the effect of patients' expectations and beliefs for acupuncturists on acupuncture efficacy, necessitating further exploration.

### Strict Quality Control Details to Help us Better Achieve Our Aim

In order to achieve our aim and have the results more reliable and replicable, we made several strict quality control details to control the quality of this trial. The details are as follows. (1) We will control the gender of the acupuncturist (both male) to exclude any potential influence of the operator's gender on the patients' expectation. (2) Acupuncturists will not be present during evaluation of clinical outcomes, to prevent excessive evaluation bias. (3) Acupuncturists have been adequately trained, and will be expected to follow the same acupuncture procedures, including acupuncture prescription, needle angle, direction, manipulation, and stimulation, among others. (4) To prevent the risk bias on the results, data collectors, outcome assessors, and data analysts will be blinded to the allocation. (5) In order to ensure compliance by all researchers, group meetings will be held every week for communication and study, and solutions will be put forward in time if difficulties or problems are encountered in the trial.

### Potential Implication of Trial Findings for Future Clinical Practice

In clinical practice, identification of practitioners who are sufficiently qualified to deliver acupuncture treatment is imperative in accurate decision making. Patients' expectations of acupuncturists or acupuncture treatment, and acupuncturists' TCM diagnosis and treatment capability, might be the key components determining who might be qualified enough to deliver acupuncture. However, these components are often mixed. Results from this trial will quantitatively clarify whether or not patients' expectations of different acupuncturists do influence acupuncture efficacy in treatment of migraine. It is possible that all the licensed acupuncturists, whether high-level or interns, might deliver similar acupuncture efficacy in migraine patients if they follow the appropriate acupuncture procedures. If this is not the case, the findings of this trial are expected to ascertain to what extent this effect size might be. We envisage that our findings will guide future acupuncture clinical trials and acupuncture clinical practice.

### Limitations

We anticipate a few limitations in the study. Firstly, although the evaluator, outcome assessor, and data analyst will be blinded, it is impossible to blind the acupuncturist due to the nature of treatment. Secondly, different acupuncturists may not achieve completely uniform acupuncture, thus both the senior and junior acupuncturist will be trained to strictly to follow the same acupuncture procedure in an attempt to minimize differences in efficacy. In addition, it is not excluded that our preliminary experimental results may underestimate the sample size and lead to false negative results.

## Ethics Statement

The studies involving human participants were reviewed and approved by Institutional Review Boards and Ethics Committees of Hospital of Chengdu University of Traditional Chinese Medicine (NO. 2020KL-058). The patients provided their written informed consent to participate in this study. This trial was registered on the Chinese Clinical Trial Registry (ChiCTR) on January 31, 2021 (Registration number: ChiCTR2100042963).

## Author Contributions

Z-jL conceived of the study. F-rL, FZ, and JZ initiated the study design. YF, H-bZ, and X-pH helped with its implementation. S-rC, JZ, N-nJ, X-lL, and S-jH prepared the informed consent and finished trial registration. JZ, N-nJ, and YF drafted the manuscript. Z-jL, F-rL, and R-rS revised the manuscript. All authors contributed to the refinement of the study protocol and approved the final manuscript.

## Funding

The trial is supported by funds from the National Natural Science Foundation of China (No.81973958), Innovation Team and Talents Cultivation Program of National Administration of Traditional Chinese Medicine (No. ZYYCXTD-D-202003), and Postdoctoral Science Foundation (Nos. 2017M610593, 2018T110954, and PC2019012). The sponsors will play no part in study design, data collection, management, and analysis. They also will not be involved in data interpretation, report writing, and the decision to submit the report for publication.

## Conflict of Interest

The authors declare that the research was conducted in the absence of any commercial or financial relationships that could be construed as a potential conflict of interest.

## Publisher's Note

All claims expressed in this article are solely those of the authors and do not necessarily represent those of their affiliated organizations, or those of the publisher, the editors and the reviewers. Any product that may be evaluated in this article, or claim that may be made by its manufacturer, is not guaranteed or endorsed by the publisher.

## Appendix

**Appendix 1**. Acupuncture Expectations Evaluation Scale (AES).

**Expectation of the acupuncturists**

Every individual may have different expectation for the acupuncturists. If we use the following sentences to describe your expectation of acupuncturists after the entire course of acupuncture treatment, how much confidence do you have? Please rate the following statements from 0 to 100, 0 being not at all, and 100 being totally cured:

“How much confidence do you have in senior acupuncturists to relieve your migraine symptoms?”

**0————–100**

“How much confidence do you have in junior acupuncturists to relieve your migraine symptoms?”

**0————–100**

**Expectation of the acupuncture therapy**

Every individual may have different expectation for the effects of acupuncture. If we use the following sentence to describe your expectation of acupuncture's effect on your illness/symptom after the entire course of acupuncture therapy, how much do you agree? Please rate the following statement from 0 to 100, 0 being not at all, and 100 being totally cured:

“How much confidence do you have in acupuncture therapy to relieve your migraine symptoms?”

**0————–100**

## References

[1] GBD 2016 Headache Collaborators. Global, regional, and national burden of migraine and tension-type headache, 1990-2016: a systematic analysis for the Global Burden of Disease Study 2016. Lancet Neurol. (2018) 17:954–76. 10.1016/S1474-4422(18)30322-3 30353868 PMC6191530

[2] Rasmussen BK Olesen J. Migraine with aura and migraine without aura: an epidemiological study. Cephalalgia. (1992) 12:22–8. 10.1046/j.1468-2982.1992.1204221.x 1525797

[3] GBD 2016 Disease and Injury Incidence and Prevalence Collaborators. Global, regional, and national incidence, prevalence, and years lived with disability for 328 diseases and injuries for 195 countries, 1990-2016: a systematic analysis for the Global Burden of Disease Study 2016. Lancet. (2017) 390:1211–59. 10.1016/S0140-6736(17)32154-2 28919117 PMC5605509

[4] All-Party Parliamentary Group on Primary Headache Disorders (APPGPHD). Headache Disorders - not respected, not resourced: A Report of the All-Party Parliamentary Group on Primary Headache Disorders (APPGPHD). (2010). Available online at: http://www.migrainetrust.org/wpcontent/uploads/2015/12/2010MarAPPGPHD_REPORT_Headache_Disorders-NotRespNotReso.pdf (accessed November 22, 2017).

[5] Burke A Upchurch DM Dye C Chyu L. Acupuncture use in the United States: findings from the National Health Interview Survey. J Altern Complement Med. (2006) 12:639–48. 10.1089/acm.2006.12.639 16970534

[6] Wells RE Bertisch SM Buettner C Phillips RS McCarthy EP. Complementary and Alternative Medicine Use among Adults with Migraines/Severe Headaches. Headache. (2011) 51:1087–97. 10.1111/j.1526-4610.2011.01917.x 21649654 PMC3627391

[7] Kristoffersen ES Grande RB Aaseth K Lundqvist C Russell MB. Management of primary chronic headache in the general population: the Akershus study of chronic headache. J Headache Pain. (2012) 13:113–20. 10.1007/s10194-011-0391-8 21993986 PMC3274574

[8] Xu SB Yu LL Luo X Wang MH Chen GH Zhang Q . Manual acupuncture versus sham acupuncture and usual care for prophylaxis of episodic migraine without aura: multicentre, randomized clinical trial. BMJ. (2020) 368:m697. 10.1136/bmj.m697 32213509 PMC7249245

[9] Zhao Ling Chen Jiao Li Ying Sun X Chang XR Zheng H . The long-term effect of acupuncture for migraine prophylaxis: a randomized clinical trial. JAMA Intern Med. (2017) 177:508–15. 10.1001/jamainternmed.2016.9378 28241154

[10] Li Z Liu M Lan L Zeng F Makris N Liang Y . Altered periaqueductal gray resting state functional connectivity in migraine and the modulation effect of treatment. Sci Rep. (2016) 6:20298. 10.1038/srep20298 26839078 PMC4738255

[11] Li Z Lan L Zeng F Makris N Hwang J Guo T . The altered right frontoparietal network functional connectivity in migraine and the modulation effect of treatment. Cephalalgia. (2017) 37:161–76. 10.1177/0333102416641665 27053062 PMC5659390

[12] Li Z Zeng F Yin T Lan L Makris N Jorgenson K . Acupuncture modulates the abnormal brainstem activity in migraine without aura patients. NeuroImage Clin. (2017) 15:367–75. 10.1016/j.nicl.2017.05.013 28580293 PMC5447510

[13] Linde K Allais G Brinkhaus B Fei YT Mehring M Vertosick EA . Acupuncture for the prevention of episodic migraine. Cochrane Database Syst Rev. (2016) 4:CD001218. 10.1002/14651858.CD001218.pub3 27351677 PMC4977344

[14] Schulz KF Altman DG Moher D CONSORT Group. CONSORT 2010 statement: updated guidelines for reporting parallel group randomized trials. Ann Intern Med. (2010) 152:726–32. 10.7326/0003-4819-152-11-201006010-00232 20335313

[15] MacPherson H Altman DG Hammerschlag R Li YP Wu TX White A . Revised standards for reporting interventions in clinical trials of acupuncture (STRICTA): extending the CONSORT statement. PLoS Med. (2010) 7:e1000261. 10.1371/journal.pmed.1000261 20543992 PMC2882429

[16] Olesen J. Headache classification committee of the International Headache Society (IHS) The International Classification of Headache Disorders, 3rd edition. Cephalalgia. (2018) 38:1–211. 10.1177/0333102417738202 29368949

[17] Hui KK Nixon EE Vangel MG Liu J Marina O Napadow V . Characterization of the “deqi” response in acupuncture. BMC Complement Altern Med. (2007) 7:33. 10.1186/1472-6882-7-33 17973984 PMC2200650

[18] Pharmacological management of migraine. Edinburgh: SIGN; 2018. (SIGN publication no. 155). Available from URL: http://www.sign.ac.uk (accessed February 2018).

[19] Tobis JM Charles A Silberstein SD Sorensen S Maini B Horwitz PA . Percutaneous closure of patent foramen ovale in patients with migraine: the PREMIUM trial. J Am Coll Cardiol. (2017) 70:2766–74. 10.1016/j.jacc.2017.09.1105 29191325

[20] Aicher B Peil H Peil B Diener HC. Pain measurement: Visual Analogue Scale (VAS) and Verbal Rating Scale (VRS) in clinical trials with OTC analgesics headache. Cephalalgia. (2012) 32:185-97. 10.1177/03331024111430856 22332207

[21] Zung WW. A rating instrument for anxiety disorders. Psychosomatics. (1971) 12:371–9. 10.1016/S0033-3182(71)71479-0 5172928

[22] Zung WW. A self-rating depression scale. Arch Gen Psychiatry. (1965) 12:63–70. 10.1001/archpsyc.1965.01720310065008 14221692

[23] Martin BC Pathak DS Sharfman MI Adelman JU Taylor F Kwong WJ. . Validity and reliability of the migraine-specific quality of life questionnaire (MSQ version 21). Headache. (2000) 40:204–15. 10.1046/j.1526-4610.2000.00030.x 10759923

[24] Fields DL. Taking the measure of work: a guide to validated scales for organizational research and diagnosis (Book). Pers Psychol. (2002) 2013:1–43. 10.4135/9781452231143.n1

[25] Stewart WF Lipton RB Whyte J Dowson A Kolodner K Liberman JN . An international study to assess reliability of the migraine disability assessment (MIDAS) score. Neurology. (1999) 53:988–94. 10.1212/WNL.53.5.988 10496257

[26] Kong J GoUub R Huang T Polich G Napadow V Hui K . Acupuncture de qi, from qualitative history to quantitative measurement. J Altern Complement Med. (2007) 13:1059–70. 10.1089/acm.2007.0524 18166116

[27] Sun XL Sun SB Chen SF Gao YB. Observation on the effect difference in migraine treated with the combination of acupuncture and blood-letting therapy and medication with carbamazepine. World J Acupunct-Moxibustion. (2021) 31:16–21. 10.1016/j.wjam.2020.10.008

[28] Han JS. Acupuncture analgesia: areas of consensus and controversy. Pain. (2011) 152:S41–8. 10.1016/j.pain.2010.10.012 21078546

[29] Vickers AJ Linde K. Acupuncture for chronic pain. JAMA. (2014) 311:955–6. 10.1001/jama.2013.285478 24595780 PMC4036643

[30] Zhao L Li D Zheng H Chang X Cui J Wang R . Acupuncture as adjunctive therapy for chronic stable angina: a randomized clinical trial. JAMA Intern Med. (2019) 179:1388–97. 10.1001/jamainternmed.2019.2407 31355870 PMC6664382

[31] Ou MQ Fan WH Sun FR Jie WX Lin MJ Cai YJ . A systematic review and meta-analysis of the therapeutic effect of acupuncture on migraine. Front Neurol. (2020) 11:596. 10.3389/fneur.2020.00596 32714268 PMC7344239

[32] Madsen MV Gotzsche PC Hrobjartsson A. Acupuncture treatment for pain: systematic review of randomized clinical trials with acupuncture, placebo acupuncture, and no acupuncture groups. BMJ. (2009) 338:a3115. 10.1136/bmj.a3115 19174438 PMC2769056

[33] Linde K Niemann K Schneider A Meissner K. How large are the nonspecific effects of acupuncture? A meta-analysis of randomized controlled trials. BMC Med. (2010) 8:75. 10.1186/1741-7015-8-75 21092261 PMC3001416

[34] MacPherson H Vertosick E Lewith G Linde K Sherman KJ Witt CM . Influence of control group on effect size in trials of acupuncture for chronic pain: a secondary analysis of an individual patient data meta-analysis. PLoS ONE. (2014) 9:e93739. 10.1371/journal.pone.0093739 24705624 PMC3976298

[35] Walwyn R Roberts C. Therapist variation within randomised trials of psychotherapy: implications for precision, internal and external validity. Stat Methods Med Res. (2010) 19:291–315. 10.1177/0962280209105017 19608603

[36] Linde K Witt CM Streng A Weidenhammer W Wagenpfeil S Brinkhaus B . The impact of patient expectations on outcomes in four randomized controlled trials of acupuncture in patients with chronic pain. Pain. (2007) 128:264–71. 10.1016/j.pain.2006.12.006 17257756

